# The efficacy, acceptability and safety of acceptance and commitment therapy for fibromyalgia – a systematic review and meta-analysis

**DOI:** 10.1177/20494637231221451

**Published:** 2023-12-12

**Authors:** Florence Eastwood, Emma Godfrey

**Affiliations:** 1 Department of Psychology, School of Mental Health & Psychological Sciences, Institute of Psychiatry, Psychology & Neuroscience, King's College London, London, UK; 2 Mid and South Essex NHS Foundation Trust, Chelmsford, UK; 3 Department of Population Health Sciences, School of Life Course & Population Sciences, Faculty of Life Sciences and Medicine, King’s College London, London, UK

**Keywords:** Chronic pain, fibromyalgia, pain, pain clinics, pain management, acceptance and commitment therapy

## Abstract

**Background:**

Fibromyalgia (FM) is a chronic pain disorder characterised by widespread pain, fatigue and cognitive symptoms. Acceptance and commitment therapy (ACT) aims to improve psychological flexibility and has been found to be beneficial in treating chronic pain; however, there are few studies evaluating its efficacy in treating FM.

**Aim:**

This systematic review and meta-analysis evaluated the efficacy of acceptance and commitment therapy in patients with fibromyalgia.

**Methods:**

PubMed, Embase and PsychInfo databases were searched. Randomised Controlled Trials (RCTs) were eligible for inclusion if participants had FM, and the intervention was based on the ACT framework/model, and not combined with any other active therapy; any non-ACT control was accepted. A meta-analysis was performed, with the primary outcomes pain acceptance (chronic pain acceptance questionnaire, CPAQ), health-related quality of life (fibromyalgia impact questionnaire, FIQ), attrition rate and frequency of adverse events, and the secondary outcomes pain intensity, disability, depression, anxiety, and fatigue.

**Results:**

Six RCTs, with a total of 384, mostly-female, participants were included, with ACT being delivered online, in a group setting, or one-to-one. ACT was superior to controls in improving FIQ score at post-intervention (SMD −1.05, 95% CI −2.02, −0.09) and follow-up (SMD −1.43, 95% CI −2.17, −0.69) and CPAQ post-intervention (SMD 1.05, 95% CI 0.61, 1.49) and at follow-up (SMD 0.95, 95% CI 0.40, 1.49). Attrition was below 20% in 4/6 studies and no adverse events were reported as attributable to ACT. All secondary outcomes showed large-to-moderate pooled effect estimates post-intervention, indicating improvement in anxiety, depression, pain and disability. Fatigue also improved, with a large negative effect.

**Conclusions:**

The results suggest ACT improved outcomes in patients with FM: there was an overall improvement in all outcomes post-intervention, with most maintained at follow-up. This review was, however, limited by the small body of evidence and differing methodologies of included studies.

## Introduction

Fibromyalgia (FM) is a chronic pain disorder characterised by widespread pain, fatigue, and cognitive symptoms.^1^ Estimates of the prevalence of FM vary between studies and countries, tending to fall between 2 and 8%, with higher rates in women than men.^2^

Fibromyalgia is associated with significant impairment and disability. One factor with a central role in many models of chronic pain and disability is an individual’s patterns of daily activity; McCracken suggests that the psychological flexibility model may help us to better understand the activity patterns of those with chronic pain, and how they link to disability.^3^

Psychological flexibility is described as ‘the ability to fully contact the present moment and the inner experiences that are occurring, without needless defence, and, depending upon the context, persisting or changing in the pursuit of goals or personal values’.^4,5^ Interventions aimed at increasing psychological flexibility have been shown in randomised controlled trials (RCTs) to be effective in treating chronic pain^6^; therefore, this review aims to explore the effectiveness of one such intervention, acceptance and commitment therapy (ACT), in fibromyalgia.

The aim of ACT is to increase psychological flexibility through six processes of change, as described by Twohig^5^: Acceptance of thoughts and feelings, including those which are negative. Cognitive defusion reduces the meaning and impact of thoughts and feelings, and the influence they have over a person’s actions. Being present involves actively paying attention to thoughts, feelings and events in the present moment, without evaluating or judging. Self as context means viewing the self as a perspective from which thoughts, feelings and events are experienced, without being defined by them. Values are principles that are important to an individual, and motivate them. Committed action put values into practice via creating and acting on goals. Many of these processes may be more useful in a particular context; in other situations, their converse may be of more use. The key is being flexible, and recognising which process is more useful in a given situation.^5^

ACT has been shown to be effective in treating chronic pain: it received strong support by Division 12 of the American Psychological Association.^7^ A systematic review of 10 RCTs of ACT for chronic pain found that, compared to inactive treatments, ACT showed a number of benefits, including small-to-large effects on physical functioning, anxiety, depression, general emotional distress and psychological flexibility, and a large effect on life satisfaction.^8^

### Aims of this systematic review and meta-analysis

This systematic review and meta-analysis will examine the efficacy and safety of acceptance and commitment therapy for FM, using the primary outcomes of pain acceptance, a component of psychological flexibility, quality of life, attrition rates and adverse events. The quality of the evidence base will be assessed, and a meta-analysis will be conducted, to combine the results of multiple studies and calculate pooled effect estimates.

## Methodology

This review was conducted according to the PRISMA (Preferred Reporting Items for Systematic Reviews and Meta-Analyses) Statement.^9^

### Protocol

Methods of analysis and inclusion/exclusion criteria were discussed in advance in meetings on 30/01/18 and 05/03/18; the protocol was specified in advance but not registered. The criteria were revised to accommodate the small evidence base, as a number of studies fit most of the criteria, but studied chronic pain rather than only fibromyalgia (FM); if the study reported the type of chronic pain, the authors were contacted to obtain the data for just the fibromyalgia patients.

### Criteria for inclusion

#### Participants

This review included studies with patients of any age or sex, with a diagnosis of FM: although a clinical diagnosis as part of the screening process was preferable, self-report was also accepted.

#### Interventions

The intervention being studied was Acceptance and Commitment Therapy (ACT). The intervention must have been based on the ACT model/framework in order for a study to be included. Studies which allowed participants to continue with their current treatment (treatment as usual, TAU), for the duration of the study, were included.

Studies in which ACT was combined with any other defined active therapy, such as physical therapy, or pharmacotherapy, with a defined treatment protocol, were excluded.

#### Types of studies and controls

This review accepted waiting list control, attention control, TAU and any other active therapy as control groups. If a study used multiple control groups, preference was given in the following order: attention control, waiting list, TAU and any other active therapy.

Studies were included if they were published in a peer-reviewed journal or available in a database. They must have been randomised controlled trials, with ACT as the active intervention, and with a trial duration of more than 2 weeks. In order to be included in this review, studies must have been written in English.

### Outcome measures

This review analysed outcome measures at the end of treatment and at follow-up. Follow-up analysis is intended to analyse the long-term effects of the intervention; therefore, follow-up data were not included if they were measured less than 3 months post-treatment.

The outcomes measured were as follows:

#### Primary outcomes

1. Pain acceptance (measured by the Chronic Pain Acceptance Questionnaire, CPAQ, or CPAQ-R)
2. Health-related quality of life (measured by the Fibromyalgia Impact Questionnaire, FIQ, or FIQR)
3. Attrition rate
4. Frequency of adverse events

#### Secondary outcomes

1. Pain
2. Disability
3. Depression
4. Anxiety
5. Fatigue

The FIQ and FIQR are questionnaires measuring the health-related quality of life of individuals with FM.^10,11^ The result is a score out of 100, with a higher score indicating a greater impact. The FIQR has a Chronbach’s alpha of 0.95.

The CPAQ-R is the revised version of the CPAQ. It is a 20-item questionnaire, measuring an individual’s pain acceptance.^12,13^ Scores are counted for two sections – pain willingness and activities engagement – and added together, giving a total score out of 120, with a higher score indicating greater pain acceptance. The Chronbach’s alpha for the CPAQ-R is 0.78.

### Study selection

#### Electronic searches

On 12/02/2018 and 26/08/2023 the following databases were searched, and the search results were collated for screening:

1. PubMed
2. Embase (1974 to February 2018; February 2018 to August 2023)
3. PsychInfo (1806 to February 2018; February 2018 to August 2023)

The following journals were manually searched: The Journal of Pain, The European Journal of Pain, Pain Practice, Pain Medicine, Journal of Pain and Symptom Management, and Pain Management Nursing.

The following search terms were used:

fibromyalgia (or) fm (or) fibromyalgia syndrome (or) fms (or) fibrositis (or) chronic widespread pain (or) cwp

(and)

acceptance and commitment therapy (or) acceptance & commitment therapy (or) act (or) comprehensive distancing (or) focused acceptance and commitment therapy (or) focused acceptance & commitment therapy (or) fact

(and)

randomised controlled trial (or) randomised control trial (or) rct (or) randomised trial (or) rt (or) randomised clinical trial (or) randomised comparative trial (or) randomized controlled trial (or) randomized control trial (or) randomized trial (or) randomized clinical trial (or) randomized comparative trial

### Measures of treatment effect

The means (M) and standard deviations (SD) were extracted from each paper for the specified outcomes into RevMan Web^14^ and used to calculate the standard mean difference (SMD) as Hedge’s *g*. The 95% confidence intervals were also calculated.

To evaluate the strength of the effect size, Cohen’s categories were used: an effect size of 0.2–0.5 may be considered small, 0.5–0.8 considered moderate and 0.8 and above considered a large effect size.^15^

### Data collection and analysis

#### Study selection

The first author (FE) screened all of the titles and abstracts, and selected studies based on the inclusion and exclusion criteria. The second author (EG) independently screened a proportion of the studies.

#### Data extraction

Data for primary and secondary outcome measures were input directly into RevMan by FE. The dropout rates and demographics of participants for each study, and the frequency of adverse events were input into a spreadsheet.

For one study, the raw data were provided by the authors. The total scores for the CPAQ-R, depression, pain intensity, and disability, were calculated for the participants with self-reported fibromyalgia, using IBM SPSS Statistics, Version 24.^16^ The means and standard deviations for baseline, post-treatment and 3-month follow-up were then calculated in the same programme, and input into the spreadsheet and RevMan. These were calculated using the same methodology as the published study.^17^

#### Synthesis of results

A meta-analysis was performed using RevMan. Data were extracted directly into the software and random-effects meta-analyses were performed for each outcome, for both post-intervention and follow-up.

#### Risk of bias

Risk of bias was assessed in accordance with the *Cochrane Handbook for Systematic Reviews of Interventions*.^18^ A risk of bias figure was produced in RevMan. Studies with 0–2 unclear or high risks of bias were deemed high-quality, those with 3–4 moderate-quality, and those with 5 were deemed low-quality, as in a systematic review on CBTs for FM.^19^

#### Assessment of treatment quality

Treatment quality was assessed using the quality rating scale by Yates and colleagues.^20^ This scores psychological interventions on treatment content/setting, duration, manualisation, adherence to the manual, therapist training and patient engagement, giving a score out of 9.

#### Additional analyses

Sub-group analyses and meta-regression analyses were planned, however not enough studies were found to give meaningful results. Sub-group analyses would compare the efficacy of different methods of delivery, and meta-regression would examine the relationship between moderator variables and the two primary outcomes, on a study level. These would also be used to explore potential sources of heterogeneity.

#### Missing data

Where a study did not report results for an outcome, its authors were contacted via email to request the missing data, either in raw form or analysed according to the study protocol. If the data were not supplied, the study was not included in the meta-analysis for that outcome.

#### Assessment of heterogeneity

In the meta-analyses, I^2^ was calculated in RevMan. I^2^ describes the proportion of variation in effect sizes that is due to heterogeneity rather than chance. If this has a low value, there is little variation between the results of the studies.

#### Assessment of publication bias

Funnel plots were generated in RevMan for the two primary outcomes, to assess the likelihood of publication bias. During the search and screening process, the author aimed to identify any study protocols for which there were no published results, indicating potential publication bias. A brief search was done of *clinicaltrials*.*gov* to find any registered trials that had not been completed.

## Results

### Study selection

**Figure 1.**
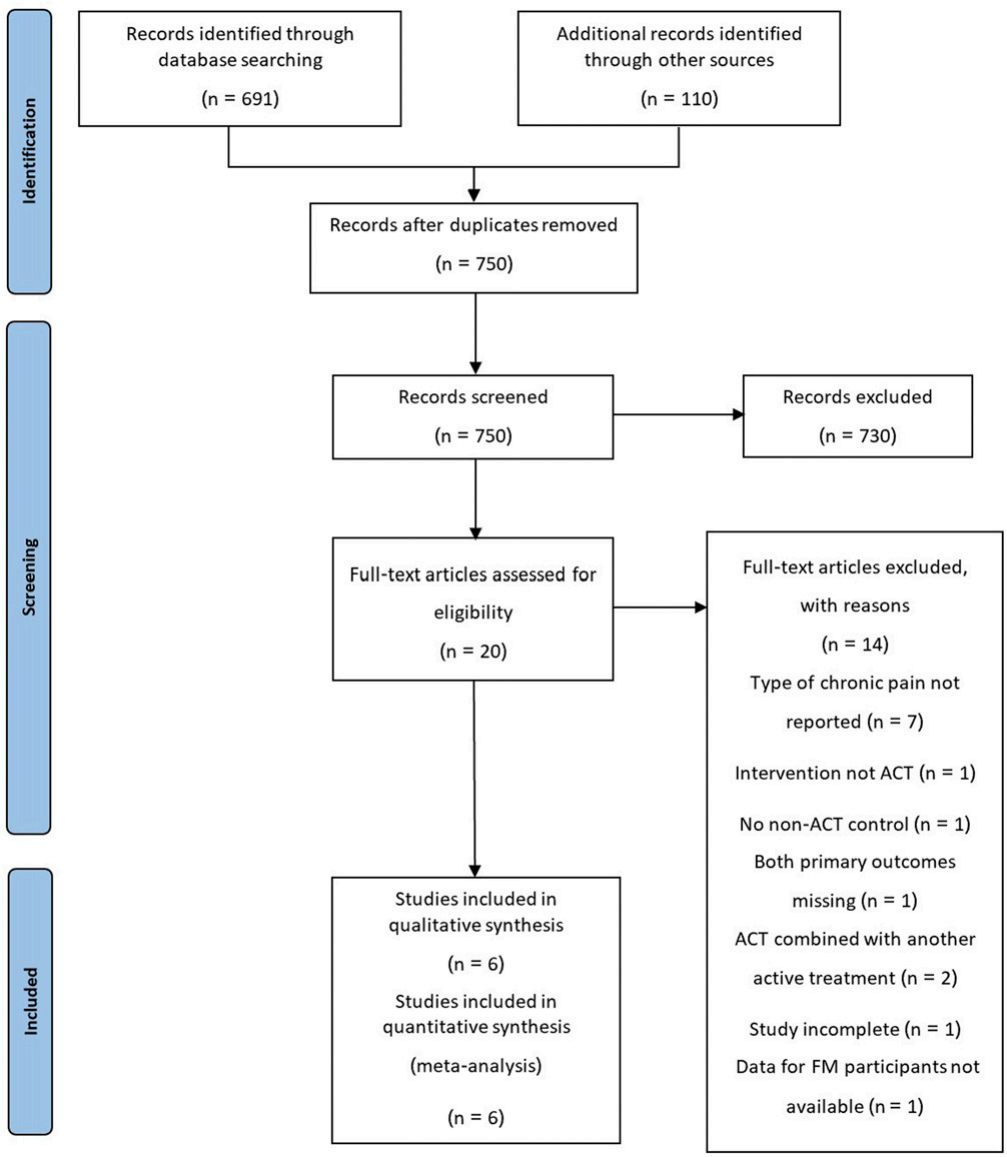
PRISMA flow diagram.

The original search and screening was done on 12/02/2018, and updated on 26/08/2023 using the same search terms and selection criteria. A total of 750 studies were identified in the searches, after duplicates were removed. Of these, 20 full-text articles were screened for eligibility, with 6 being suitable for inclusion (Table 1; Figure 1).^17,21–25^ The remaining 14 were excluded for the following reasons: 7 did not report the type of chronic pain,^26–32^ 1 had no ACT intervention,^33^ 1 had no non-ACT control,^34^ 1 did not report either primary outcome,^35^ 2 combined ACT with another active treatment,^36,37^ 1 was an incomplete study,^38^ and 1 had no available data for FM participants.^39^

**Table 1.** Characteristics of included studies.

Author(s)	Year	Country	Total *n*	Mean age, y (SD)	Female (%)	Intervention (*n*)	Control (*n*)
Catella et al.^25^	2023	USA	67	ACT = 52.2 (10.51); ST = 53.6 (10.14)	98.5	OACT^a^ (*n* = 39)	ST (*n* = 28)
Luciano et al.^21^	2014	Spain	156	GACT = 48.9 (5.94); RPT = 47.77 (5.87); WL = 48.3 (5.71)	96.2	GACT (*n* = 51)	RPT (*n* = 52); WL (*n* = 53)
McCracken et al.^17^	2013	UK	26	55.6 (11.46)	92.3	GACT+TAU (*n* = 12)	TAU (*n* = 14)
Simister et al.^22^	2018	Canada	67	39.7 (9.36)	95.0	OACT+TAU (*n* = 33)	TAU (*n* = 34)
Steiner^23^	2013	USA	33	48.6 (12.96)	100.0	1-1 ACT (*n* = 18)	ED (*n* = 15)
Wicksell et al.^24^	2013	Sweden	40	45.1 (6.60)	100.0	GACT (*n* = 23)	WL (*n* = 17)

GACT: group ACT; OACT: online ACT; TAU: treatment as usual; ST: symptom tracking; RPT: recommended pharmacological treatment; WL: waitlist; ED: education.

^a^OACT in this study was delivered via a smartphone application.

The authors of two studies provided data that were not available in the full-text publications: McCracken et al provided data for the participants with fibromyalgia, as outlined in the methodology for this review^17^; Catella et al. provided the baseline and post-intervention data for FIQ-R, pain intensity and depression, as mean scores with standard deviation, via email.^25^ The authors for one paper were contacted to obtain data for FIQ, disability status and any follow-up data, however, this data was not available.^23^

### Study characteristics

### Settings and recruitment

Three studies were conducted in North America (two in the USA and one in Canada) and three in Europe (Spain, United Kingdom and Sweden). Patients were recruited/referred from primary care, local clinics and self-help groups, by advertisements online, in flyers and in local newspapers, and via study site internal databases.

Five studies recruited via referrals from physicians, three of which recruited from primary care centres. Other recruitment methods were: self-referral from local clinics and self-help groups, via advertisements in local newspapers, digital advertising and flyers, and recruitment via internal databases at the study site.

### Study design

Three of the six studies included were pilot studies.^17,23,25^ Three used a computer-generated randomisation sequence to randomise participants,^17,21,22^ one used dynamic randomisation^25^ and one had an independent researcher, who was not involved in the trial, conduct randomisation using sealed envelopes containing codes for each study arm.^24^ One study used a coin-flip method for randomisation.^23^

Two studies used the ACR 1990 criteria to confirm diagnosis of fibromyalgia,^21,24^ one used the ACR 2010 criteria,^22^ and one used the ACR 2016 criteria.^25,40–42^ One study required a physician diagnosis of FM, not using a specific criteria,^23^ and one study required persistent pain for more than 3 months, and a pain-related consultation with a primary care physician in the last 6 months, for inclusion, with diagnoses being self-reported.^17^ Two studies also required a minimum FIQ/FIQ-R score for inclusion,^23,25^ one required a minimum score rating the interference with daily activities due to pain,^17^ and a further two required a minimum pain intensity score for inclusion.^22,24^

Five studies excluded severe psychiatric comorbidities, including active suicidality, psychosis, untreated major depression, alcohol or substance misuse and cognitive impairment.^21–25^ Five studies excluded comorbid somatic or rheumatic conditions, or any medical condition that may interfere with the study.^6,7,21–23,25^ Two studies required participants to either take no pharmacological treatment for their FM, or to discontinue it for the duration of the trial,^21,24^ and three studies excluded those currently or recently receiving psychological treatments.^21,24,25^ Some form of psychiatric assessment/interview was carried out in three studies.^21,23,24^ One study used functional magnetic resonance imaging during the research project, therefore excluded left-handed, pregnant or breastfeeding participants, and those with metal implants or claustrophobia.^24^

Three studies had a follow-up period of 3 months post-intervention,^17,22,24^ and one study had a 6 month follow-up period.^21^ One study followed up participants after 12 weeks, but did not report follow-up results, and the author was contacted.^23^ One study did not report follow-up.^25^

### Participants

The included studies had a total of 384 participants: 176 in the intervention groups and 208 in the control groups. The median mean age of participants was 48.75 and the median proportion of participants who were women was 97.4%.

### Interventions

One study gave eight weekly sessions of one-to-one ACT, each lasting an hour.^23^ Three used a group setting to deliver ACT (GACT): Luciano et al used eight sessions, each 2.5 h long, with 10–15 per group^21^; McCracken et al delivered four 4-h sessions over 2 weeks, with 12–13 per group^17^; Wicksell et al delivered 12 weekly sessions, each 90 min, with 6 participants per group.^24^ Two studies used online ACT (OACT): Catella et al delivered a total of 41 self-guided sessions over 12 weeks via a smartphone app,^25^ and Simister et al delivered 7 online modules via an online platform, giving participants 8 weeks to complete the modules in their own time.^22^

### Controls

The control conditions used varied between studies. One study used digital symptom tracking via a smartphone app,^25^ another gave education sessions on FM.^23^ Two used treatment as usual (TAU),^17,22^ and one used a waitlist control group with no active treatment.^24^ One study had two non-ACT groups: waitlist and recommended pharmacological treatment, with pregabalin 300–600 mg daily with the addition of duloxetine 60–120 mg daily if major depression was diagnosed.^21^ Three studies offered their control groups access to the ACT intervention after completion of the study.^21,22,24^

### Reported treatment quality

The interventions were scored according to the treatment quality rating scale. A score of 0–2 was considered as indicating poor, 3–5 as average and 6–9 as excellent, as per Bernardy et al.^20,43^ All six studies had a high treatment quality: five studies scored 8/9 on the scale^17,21,22,24,25^ and one scored 7/9.^23^ Points were lost for either reported training of therapists or evidence of participant engagement in the intervention.

### Risk of bias and conflict of interest

**Figure 2.**
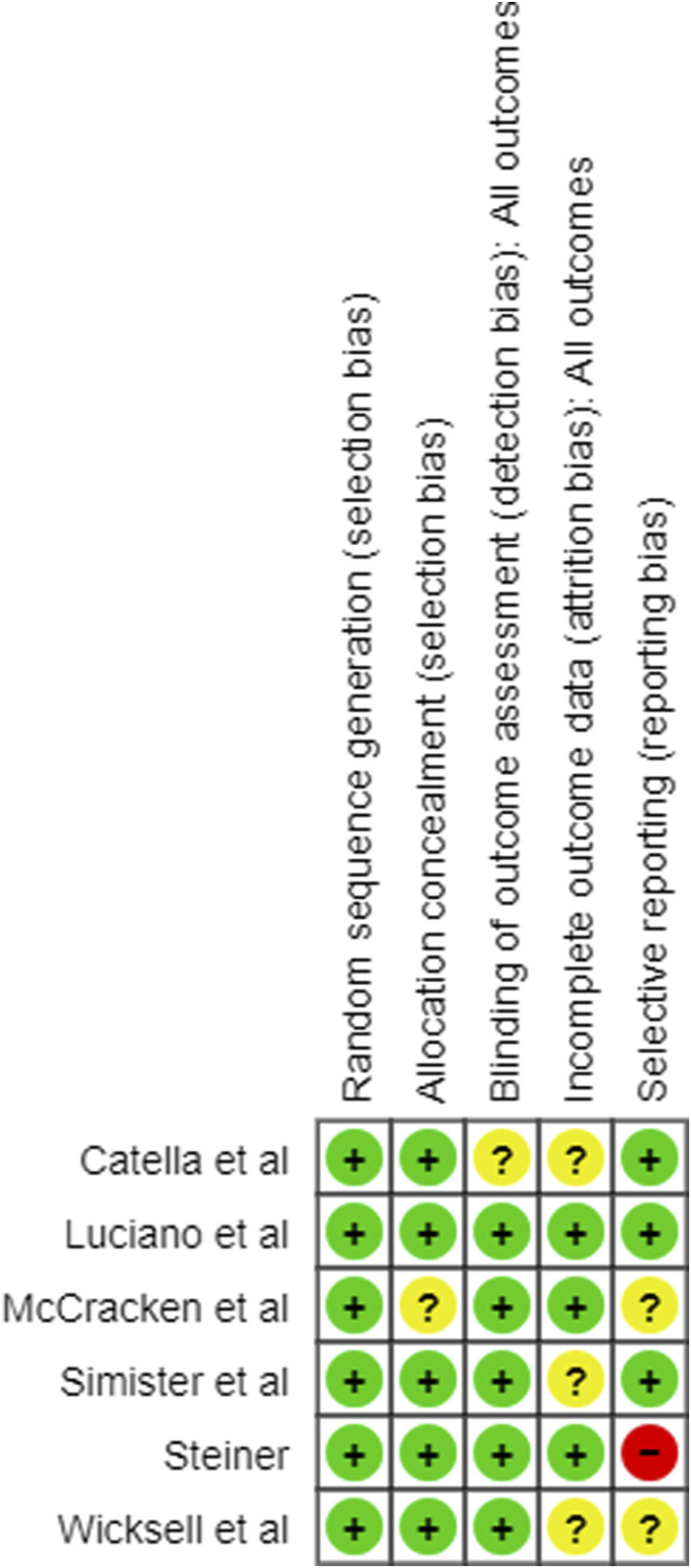
Risk of bias summary.

Of the six studies analysed, all had 0–2 unclear or high risk of bias, and are therefore deemed high quality (Figure 2). Blinding of participants to their allocation was not considered, due to the nature of the intervention.

One study reported potential conflict of interest, with many of the authors being paid consultants, shareholders and/or employees of the company which produced the app used to administer ACT, and funded the study.^25^ No other studies reported conflict of interest.

### Results of individual studies

#### Power

Statistical power was calculated, using the primary outcomes of FIQ and CPAQ scores at post-treatment assessment, with an online calculator,^44^ and the lowest values reported. Three studies had statistical power of greater than 0.80 (1.00, 0.93 and 0.89, respectively),^21–23^ with the remaining three having statistical power of 0.32,^17^ 0.26^24^ and 0.16.^25^

#### Primary outcomes

**Figure 3.**
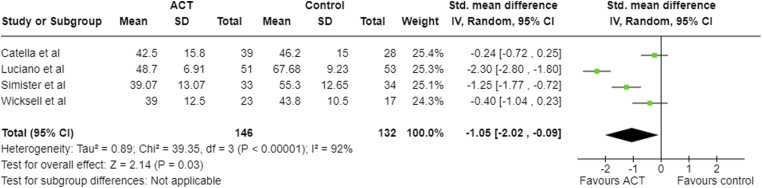
Meta-analysis results and forest plot for FIQ post-intervention.

Four studies reported FIQ/FIQ-R scores at post-treatment (Table 2; Figure 3).^21,22,24,25^ One study measured FIQ but did not report the results.^23^ Effect sizes for the FIQ, at post-intervention and follow-up, were large for two studies,^21,22^ and small for two.^24,25^ These were negative, indicating decrease in FIQ score, thus improved quality of life, favouring ACT.

**Table 2.** Effect sizes for primary outcomes in each included study.

Author(s)		FIQ/FIQ-R	CPAQ/CPAQ-R
Catella et al.^25^	Post	−0.24 (−0.72, 0.25)	—
	Follow-up	—	—
Luciano et al.^21^	Post	−2.30 (−2.80, −1.80)	1.52 (1.08, 1.96)
	Follow-up	−1.99 (−2.46, −1.52)	1.40 (0.97, 1.84)
McCracken et al.^17^	Post	—	0.55 (−0.24, 1.34)
	Follow-up	—	0.46 (−0.32, 1.25)
Simister et al.^22^	Post	−1.25 (−1.77, −0.72)	0.82 (0.32, 1.32)
	Follow-up	−1.56 (−2.11, −1.01)	0.79 (0.29, 1.28)
Steiner^23^	Post	—	1.10 (0.36, 1.85)
	Follow-up	—	—
Wicksell et al.^24^	Post	−0.40 (−1.04, −0.23)	—
	Follow-up	−0.65 (−1.30, −0.01)	—

Effect size given as SMD (95% confidence intervals).

FIQ: fibromyalgia impact questionnaire, CPAQ: chronic pain acceptance questionnaire.

**Figure 4.**
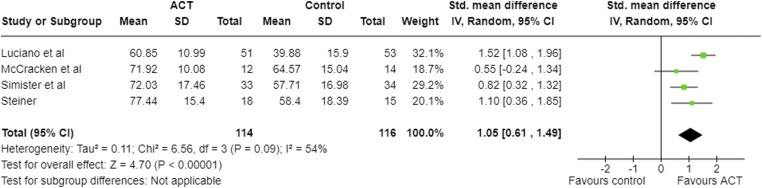
Meta-analysis results and forest plot for CPAQ post-intervention.

Four studies reported pain acceptance, using the CPAQ/CPAQ-R, at post-treatment (Table 2; Figure 4),^17,21–23^ with all but one of these studies reporting results at follow-up.^23^ Effect sizes were large for three studies,^21–23^ and moderate for one at post-treatment,^17^ with one study maintaining a large effect at follow-up.^21^ These effect sizes were positive, indicating increase in pain acceptance, favouring ACT.

One of the included studies included recommended pharmacological treatment (RPT) as a non-ACT arm.^21^ For GACT versus RPT, effect sizes were −1.80 (−2.26, −1.34) for FIQ post-treatment, and −1.76 (−2.21, −1.30) at 6-month follow-up, both in favour of GACT. For CPAQ, effect sizes were 1.35 (0.92, 1.78) post-treatment and 1.16 (0.75, 1.58) at follow-up.

#### Secondary outcomes

All six studies reported pain intensity (Table 3). Two studies used a pain visual analogue scale (PVAS),^21,23^ three used a numerical rating from 0–10,^17,24,25^ and one used the short-form of the McGill Pain Questionnaire.^22,45^

**Table 3.** Effect sizes for secondary outcomes in each included study.

Author(s)		Pain	Disability	Depression	Anxiety
Catella et al.^25^	Post	−0.21 (−0.69, 0.28)	—	0.07 (−0.42, 0.55)	—
	Follow-up	—	—	—	—
Luciano et al.^21^	Post	−1.20 (−1.62, −0.78)	—	−1.85 (−2.32, −1.39)	−0.96 (−1.37, −0.55)
	Follow-up	−1.10 (−1.51, −0.68)	—	−1.41 (−1.85, −0.98)	−1.02 (−1.43, −0.61)
McCracken et al.^17^	Post	−0.47 (−1.26, 0.31)	−0.33 (−1.10, 0.45)	−0.93 (−1.74, −0.11)	—
	Follow-up	0.24 (−0.53, 1.02)	−0.22 (−0.99, 0.56)	−0.48 (−1.26, −0.31)	—
Simister et al.^22^	Post	−0.83 (−1.33, −0.33)	—	−0.86 (−1.36, −0.35)	—
	Follow-up	−0.11 (−0.59, 0.37)	—	−0.55 (−1.04, −0.06)	—
Steiner^23^	Post	−0.90 (−1.62, −0.18)	—	—	—
	Follow-up	—	—	—	—
Wicksell et al.^24^	Post	−0.34 (−0.97, 0.29)	−0.73 (−1.38, −0.08)	−0.45 (−1.08, 0.19)	−0.72 (−1.37, −0.07)
	Follow-up	−0.80 (−1.46, −0.15)	−0.71 (−1.36, −0.06)	−0.63 (−1.27, 0.02)	−0.73 (−1.38, −0.08)

Effect size given as SMD (95% confidence intervals).

Two studies reported disability; McCracken and colleagues used the Roland and Morris Disability Questionnaire,^17,46^ whereas Wicksell and colleagues used the Pain Disability Index.^24,47^

Five studies reported depression, with Luciano and colleagues using the Hospital Anxiety and Depression Scale (HADS),^21,48^ McCracken and colleagues the Patient Health Questionnaire-9,^17,49^ Simister and colleagues the Centre for Epidemiologic Studies Depression Scale,^22,50^ and Wicksell and colleagues, and Catella and colleagues, the Beck Depression Inventory (BDI) and BDI-II, respectively.^24,25,51,52^

Anxiety was reported by two studies: Luciano and colleagues used the HADS, and Wicksell and colleagues the Spielberger Trait-State Anxiety Inventory – this review used the trait anxiety score.^21,24,48,53^

Fatigue was reported by one study,^23^ measured using the Brief Fatigue Inventory,^54^ with an SMD of −0.94 (−1.67 to −0.22).

For GACT versus RPT,^23^ SMD for pain was −0.83 (−1.24, −0.43) and −0.60 (−1.00, −0.20) at post-treatment and follow-up, respectively. For depression, SMD was −0.92 (−1.33, −0.51) and −0.72 (−1.12, −0.32); for anxiety, SMD was −0.34 (−0.73, 0.05) and −0.42 (−0.81, −0.03), all in favour of GACT.

#### Attrition and adverse events

According to Dumville and colleagues, attrition rates between 5 and 20% may be a potential source of bias, and over 20% poses a greater risk of bias.^55^ One study had attrition rates over 20% (Table 4)^23^; the control group had 40.0% attrition, whereas the intervention group had 16.7%, making the overall attrition rate 27.3%. All other included studies had attrition rates between 5 and 10%.

**Table 4.** Attrition rates between randomisation and follow-up.

Study	Total	Intervention	Control
Catella et al.^25^	13.4	15.4	10.7
Luciano et al.^21^	12.8	11.8	11.3
McCracken et al.^17^	15.4	16.7	14.3
Simister et al.^22^	16.4	15.2	17.6
Steiner^23^	27.3	16.7	40.0
Wicksell et al.^24^	17.5	17.4	17.6

Given as percentages (%).

No studies reported adverse events relating to the ACT intervention.

#### Meta-analysis

Post-intervention, large negative pooled effect sizes were seen in FIQ (Figure 3), CPAQ (Figure 4), depression and anxiety, and a large positive effect in pain acceptance (Table 5). Moderate effect sizes were seen in pain and disability. None of the 95% confidence intervals for the pooled effect sizes included zero.

**Table 5.** Meta-analysis results post-intervention for all outcomes.

Outcome	SMD_pooled_	95% CI_pooled_	df	I^2^ (%)	Z	*p*
FIQ	−1.05	−2.02, −0.09	3	92	2.14	.03
CPAQ	1.05	0.61, 1.49	3	54	4.70	<.00001
Pain	−0.68	−1.03, −0.33	5	56	3.81	.0001
Disability	−0.56	−1.06, −0.07	1	0	2.22	.03
Depression	−0.81	−1.53, −0.09	4	88	2.20	.03
Anxiety	−0.89	−1.24, −0.55	1	0	5.08	<.00001

Pooled effect sizes given as SMD_pooled_ with pooled 95% confidence intervals.

df: degrees of freedom.

**Figure 5.**
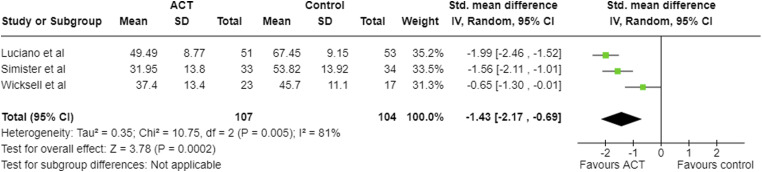
Meta-analysis results and forest plot for FIQ follow-up.

**Figure 6.**
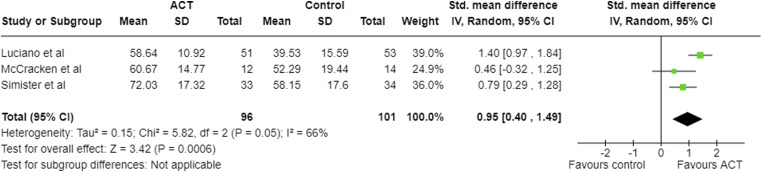
Meta-analysis results and forest plot for CPAQ at follow-up.

At follow-up, large negative pooled effect sizes were seen in FIQ (Figure 5), CPAQ (Figure 6), depression and anxiety, and a large positive effect in pain acceptance (Table 6). For disability, the pooled effect size was negative and moderate, however, the upper 95% confidence interval was −0.01. For pain intensity at follow-up, the pooled SMD was −0.48; however, the upper limit of the 95% confidence intervals was greater than zero. Therefore, there may be no effect or even a positive effect.

**Table 6.** Meta-analysis results at follow-up for all outcomes.

Outcome	SMD_pooled_	95% CI_pooled_	df	I^2^ (%)	Z	*p*
FIQ	−1.43	−2.17, −0.69	2	81	3.78	.0002
CPAQ	0.95	0.40, 1.49	2	66	3.42	.0006
Pain	−0.48	−1.09, 0.14	3	79	1.52	.13
Disability	−0.51	−1.00, −0.01	1	0	2.00	.05
Depression	−0.81	−1.31, −0.31	3	68	3.18	.001
Anxiety	−0.94	−1.29, −0.59	1	0	5.32	<.00001

Pooled effect sizes given as SMD_pooled_ with pooled 95% confidence intervals.

df: degrees of freedom.

#### Risk of bias across studies

**Figure 7.**
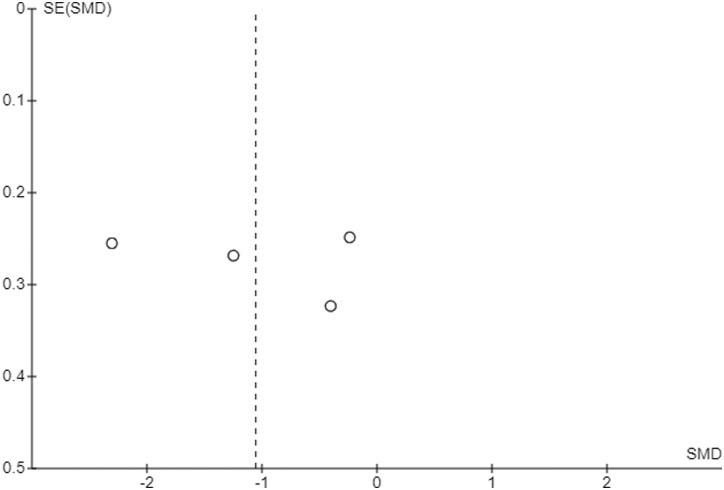
Funnel plot for FIQ at post-intervention.

Funnel plots were produced for the two primary outcomes, FIQ and CPAQ, at post-intervention, which did not indicate a high risk for publication bias (Figure 7).

A search of *clinicaltrials*.*gov* found four registered RCTs with no results published. One was listed as completed on 15/05/2023 (NCT05243511), one as ongoing (NCT05011162), one as not yet recruiting (NCT05962437), and a further trial with unknown status (NCT01611831).

## Discussion

Six studies were included in this review. With regards to the primary outcomes – health-related quality of life (FIQ/FIQR) and pain acceptance (CPAQ/CPAQ-R) – ACT was superior to controls (WL, TAU, and ED), with a large negative pooled effect (SMD −1.05, 95% CI −2.02, −0.09) for FIQ at post-intervention, a large negative pooled effect (SMD −1.43, 95% CI -2.17, −0.69) at follow-up, and large positive pooled effect for CPAQ at post-intervention (SMD 1.05, 95% CI 0.61, 1.49) and follow-up (SMD 0.95, 95% CI 0.40, 1.49). In one study, ACT was also superior to recommended pharmacological treatment (SMD at post-treatment −1.80 (−2.26, −1.34) for FIQ and 1.35 (0.92, 1.78) for CPAQ).^21^ These results indicate improvement in both pain acceptance and health-related quality of life, which was maintained at follow-up. FIQ and CPAQ, however, were each only measured by four out of six studies.

For the secondary outcomes, all showed moderate-to-large pooled effect estimates at post-intervention, indicating improvement in anxiety, depression, pain, and disability. Fatigue also showed improvement, with a large negative effect. At follow-up, depression and anxiety showed moderate and large pooled effect estimates, respectively; however, for pain intensity, the 95% confidence intervals included zero; therefore, the pooled effect estimate is not statistically meaningful. For disability and depression at post-intervention, and for disability at follow-up, the 95% confidence intervals were close to zero.

Only one study had greater than 20% attrition,^23^ although, upon further examination, this was due to high attrition in the control group, and not the intervention group; this poses a risk of bias in the results. No adverse events were reported that could be attributed to either the intervention or control conditions.

Heterogeneity was high for several outcomes in the meta-analysis, with I^2^ ranging from 0 to 92%; however, potential sources of heterogeneity could not be explored fully, as there were not enough studies to produce meaningful results in sub-group analysis or meta-regression. Across the six studies, four different modes of delivery of the intervention were used: three studies used group ACT,^17,21,24^ two used online ACT,^22,25^ one of which used a smartphone application to deliver ACT,^25^ and one study used one-on-one ACT.^23^ There were not enough studies in this review to meaningfully determine the presence or absence of publication bias. Four incomplete trials were found registered on *clinicaltrials*.*gov*, three of which were recently registered and ongoing, and a further being of unknown status.

This review has considerable limitations. Reviewing such a small body of evidence meant that inclusion/exclusion criteria could not be as strict as in a larger scale review, thus the studies included differed in their methodologies, sample sizes and outcome measures. Three out of six studies included had fewer than 50 participants (with FM) in total,^17,23,24^ and three studies were underpowered.^17,24,25^ Although Hedges *g* corrects this to a degree, small samples can lead to overestimation of effect size, giving the results an upwards bias, and meaning that the true effect sizes may be lower than those calculated in this review.

The studies that were reviewed took place in Europe and North America, in a number of different settings, including primary care, making the results of this review generalisable to clinical practice, although it is unclear whether the results are applicable outside of Europe and North America.

ACT – online, group and one-to-one – may be effective in improving outcomes for those with FM. The efficacy, acceptability and safety of ACT in FM requires further investigation with larger scale RCTs, using similar methodology and outcome measures. This would allow sub-group analysis comparing the efficacy of the different modes of delivery. Use of different modes of delivery of ACT has implications for those with access barriers, for example, financial or physical, which may prevent them from attending in-person ACT sessions. The cost-effectiveness of the different modes of delivery could also be compared.

Fibromyalgia is a condition associated with significant impairment and disability, with current pharmacotherapy having only modest effects,^56^ as well as side effects which often mimic or worsen other symptoms of FM, such as fatigue.^57^ This review, therefore, has important clinical implications, as the results show an overall improvement in all outcomes at post-intervention, with most improvements maintained at follow-up; these include pain, quality of life, disability, depression, anxiety, and fatigue. One study in this review found ACT to be superior to recommended pharmacological treatment,^21^ suggesting that use of this intervention in clinical practice for the treatment of FM may reduce the need for pharmacotherapy. Future research should explore this further, by investigating the efficacy of ACT versus pharmacotherapy, and combined with pharmacotherapy.

To conclude, this systematic review and meta-analysis has found acceptance and commitment therapy to be effective in improving a number of outcomes in individuals with fibromyalgia. It addresses important limitations in the current body of research, including the small number of studies, differing methodologies, and small samples, and suggests future direction for this field. Larger scale RCTs, comparing ACT with active control treatments as well as inactive controls, are required. Sub-group analyses, investigating moderator variables, and comparing modes of delivery, outcomes in male and female patients, and in different countries, will help us to understand the effects of ACT on FM.

## Supplemental Material


Supplemental Material - The efficacy, acceptability and safety of acceptance and commitment therapy for fibromyalgia – a systematic review and meta-analysis
Supplemental Material for The efficacy, acceptability and safety of acceptance and commitment therapy for fibromyalgia – a systematic review and meta-analysis by Florence Eastwood and Emma Godfrey in British Journal of Pain.
