# Supplementary material for: The efficacy, acceptability and safety of acceptance and commitment therapy for fibromyalgia – a systematic review and meta-analysis
Source: Br J Pain. 2023 Dec 12;18(3):243–56. doi: 10.1177/20494637231221451 (PMC11092929; doi:10.1177/20494637231221451)
Supplement: Supplemental Material - The efficacy, acceptability and safety of acceptance and commitment therapy for fibromyalgia – a systematic review and meta-analysis [file sj-pdf-1-bjp-10.1177_20494637231221451.pdf]

Study or Subgroup	ACT Mean	ACT SD	ACT Total	Control Mean	Control SD	Control Total	Weight	Std. mean difference IV, Random, 95% CI
Catella et al	5.3	1.9	39	5.7	1.9	28	19.2%	-0.21 [-0.69 , 0.28]
Luciano et al	48.07	10.5	51	64.28	15.76	53	21.3%	-1.20 [-1.62 , -0.78]
McCracken et al	6.61	1.44	12	7.38	1.69	14	12.1%	-0.47 [-1.26 , 0.31]
Simister et al	13.8	8.81	33	21	8.41	34	18.8%	-0.83 [-1.33 , -0.33]
Steiner	5	2.1	18	6.91	2.03	15	13.3%	-0.90 [-1.62 , -0.18]
Wicksell et al	4	1.1	23	4.4	1.2	17	15.3%	-0.34 [-0.97 , 0.29]
**Total (95% CI)**			**176**			**161**	**100.0%**	**-0.68 [-1.03 , -0.33]**

Heterogeneity: Tau² = 0.10; Chi² = 11.40, df = 5 (P = 0.04); I² = 56%
Test for overall effect: Z = 3.81 (P = 0.0001)
Test for subgroup differences: Not applicable

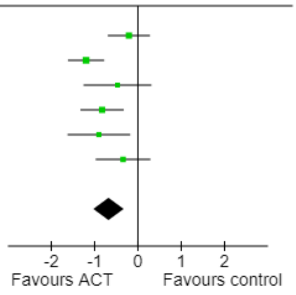

Supp Figure1. Pain post-intervention

Study or Subgroup	ACT Mean	ACT SD	ACT Total	Control Mean	Control SD	Control Total	Weight	Std. mean difference IV, Random, 95% CI
McCracken et al	11.83	4.21	12	13.5	5.5	14	41.1%	-0.33 [-1.10 , 0.45]
Wicksell et al	27.2	13.2	23	37.8	15.6	17	58.9%	-0.73 [-1.38 , -0.08]
**Total (95% CI)**			**35**			**31**	**100.0%**	**-0.56 [-1.06 , -0.07]**

Heterogeneity: Tau² = 0.00; Chi² = 0.61, df = 1 (P = 0.44); I² = 0%
Test for overall effect: Z = 2.22 (P = 0.03)
Test for subgroup differences: Not applicable

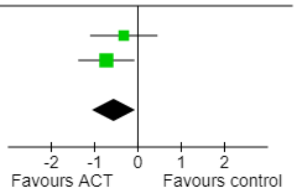

Supp Figure 2. Disability post-intervention

Study or Subgroup	ACT Mean	ACT SD	ACT Total	Control Mean	Control SD	Control Total	Weight	Std. mean difference IV, Random, 95% CI
Catella et al	11.8	7.6	39	11.3	7.5	28	20.9%	0.07 [-0.42 , 0.55]
Luciano et al	5.41	1.36	51	9.34	2.63	53	21.1%	-1.85 [-2.32 , -1.39]
McCracken et al	12	6.12	12	18.43	7.19	14	17.8%	-0.93 [-1.74 , -0.11]
Simister et al	17.76	10.83	33	26.97	10.46	34	20.7%	-0.86 [-1.36 , -0.35]
Wicksell et al	11.7	6	23	14.8	7.8	17	19.6%	-0.45 [-1.08 , 0.19]
**Total (95% CI)**			**158**			**146**	**100.0%**	**-0.81 [-1.53 , -0.09]**

Heterogeneity: Tau² = 0.58; Chi² = 33.32, df = 4 (P < 0.00001); I² = 88%
Test for overall effect: Z = 2.20 (P = 0.03)
Test for subgroup differences: Not applicable

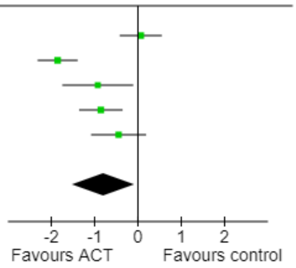

Supp Figure 3. Depression post-intervention

Study or Subgroup	ACT Mean	ACT SD	ACT Total	Control Mean	Control SD	Control Total	Weight	Std. mean difference IV, Random, 95% CI
Luciano et al	8.28	2.38	51	11.36	3.8	53	71.8%	-0.96 [-1.37 , -0.55]
Wicksell et al	40.6	10.4	23	49.3	13.5	17	28.2%	-0.72 [-1.37 , -0.07]
**Total (95% CI)**			**74**			**70**	**100.0%**	**-0.89 [-1.24 , -0.55]**

Heterogeneity: Tau² = 0.00; Chi² = 0.37, df = 1 (P = 0.54); I² = 0%
Test for overall effect: Z = 5.08 (P < 0.00001)
Test for subgroup differences: Not applicable

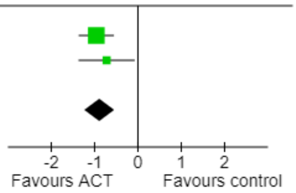

Supp Figure 4. Anxiety post-intervention

Study or Subgroup	ACT Mean	ACT SD	ACT Total	Control Mean	Control SD	Control Total	Weight	Std. mean difference IV, Random, 95% CI
Luciano et al	49.58	10.98	51	64.36	15.34	53	28.2%	-1.10 [-1.51 , -0.68]
McCracken et al	7.06	1.31	12	6.64	1.91	14	21.3%	0.24 [-0.53 , 1.02]
Simister et al	21.46	9.1	33	22.49	9.21	34	27.0%	-0.11 [-0.59 , 0.37]
Wicksell et al	3.9	1.1	23	4.8	1.1	17	23.6%	-0.80 [-1.46 , -0.15]
**Total (95% CI)**			**119**			**118**	**100.0%**	**-0.48 [-1.09 , 0.14]**

Heterogeneity: Tau² = 0.30; Chi² = 14.46, df = 3 (P = 0.002); I² = 79%
Test for overall effect: Z = 1.52 (P = 0.13)
Test for subgroup differences: Not applicable

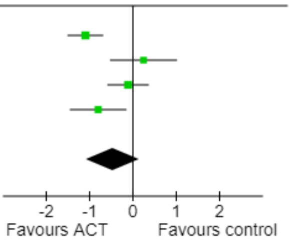

Supp Figure 5. Pain at follow-up

Study or Subgroup	ACT Mean	ACT SD	ACT Total	Control Mean	Control SD	Control Total	Weight	Std. mean difference IV, Random, 95% CI
McCracken et al	13	4.84	12	14.07	4.68	14	41.2%	-0.22 [-0.99 , 0.56]
Wicksell et al	28.1	12.5	23	38.1	15.4	17	58.8%	-0.71 [-1.36 , -0.06]
**Total (95% CI)**			**35**			**31**	**100.0%**	**-0.51 [-1.00 , -0.01]**

Heterogeneity: Tau² = 0.00; Chi² = 0.91, df = 1 (P = 0.34); I² = 0%
Test for overall effect: Z = 2.00 (P = 0.05)
Test for subgroup differences: Not applicable

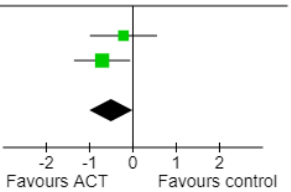

Supp Figure 6. Disability at follow-up

Study or Subgroup	ACT Mean	ACT SD	ACT Total	Control Mean	Control SD	Control Total	Weight	Std. mean difference IV, Random, 95% CI
Luciano et al	5.84	1.6	51	9.32	3.04	53	29.5%	-1.41 [-1.85 , -0.98]
McCracken et al	13.91	5.8	12	16.86	6.19	14	19.5%	-0.48 [-1.26 , 0.31]
Simister et al	18.36	12.12	33	25.13	12.29	34	27.8%	-0.55 [-1.04 , -0.06]
Wicksell et al	10.7	4.8	23	16.4	12.5	17	23.2%	-0.63 [-1.27 , 0.02]
**Total (95% CI)**			**119**			**118**	**100.0%**	**-0.81 [-1.31 , -0.31]**

Heterogeneity: Tau² = 0.17; Chi² = 9.26, df = 3 (P = 0.03); I² = 68%
Test for overall effect: Z = 3.18 (P = 0.001)
Test for subgroup differences: Not applicable

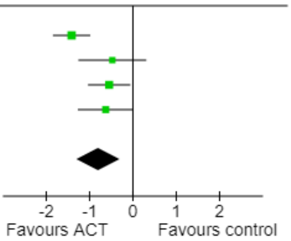

Supp Figure 7. Depression at follow-up

Study or Subgroup	ACT Mean	ACT SD	ACT Total	Control Mean	Control SD	Control Total	Weight	Std. mean difference IV, Random, 95% CI
Luciano et al	8.73	2.04	51	12.15	4.2	53	71.5%	-1.02 [-1.43 , -0.61]
Wicksell et al	39.9	9.8	23	47.9	11.8	17	28.5%	-0.73 [-1.38 , -0.08]
**Total (95% CI)**			**74**			**70**	**100.0%**	**-0.94 [-1.29 , -0.59]**

Heterogeneity: Tau² = 0.00; Chi² = 0.54, df = 1 (P = 0.46); I² = 0%
Test for overall effect: Z = 5.32 (P < 0.00001)
Test for subgroup differences: Not applicable

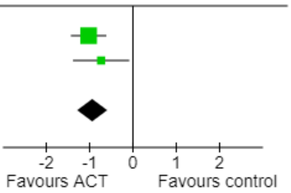

Supp Figure 8. Anxiety at follow-up

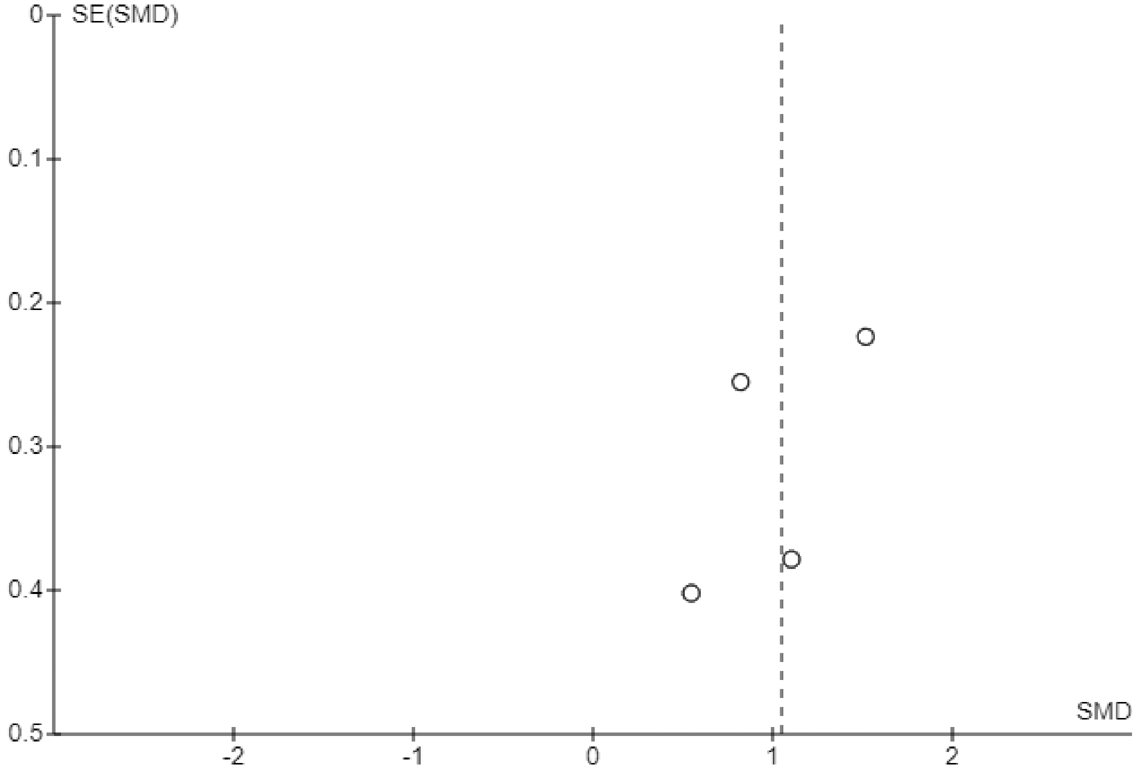

Supp Figure 9. Funnel plot for CPAQ post-intervention
